# Variable Selection in ROC Regression

**DOI:** 10.1155/2013/436493

**Published:** 2013-11-07

**Authors:** Binhuan Wang

**Affiliations:** New York University School of Medicine, New York, NY 10016, USA

## Abstract

Regression models are introduced into the *receiver operating characteristic* (ROC) analysis to accommodate effects of covariates, such as genes. If many covariates are available, the variable selection issue arises. The traditional induced methodology separately models outcomes of diseased and nondiseased groups; thus, separate application of variable selections to two models will bring barriers in interpretation, due to differences in selected models. Furthermore, in the ROC regression, the accuracy of area under the curve (AUC) should be the focus instead of aiming at the consistency of model selection or the good prediction performance. In this paper, we obtain one single objective function with the group SCAD to select grouped variables, which adapts to popular criteria of model selection, and propose a two-stage framework to apply the focused information criterion (FIC). Some asymptotic properties of the proposed methods are derived. Simulation studies show that the grouped variable selection is superior to separate model selections. Furthermore, the FIC improves the accuracy of the estimated AUC compared with other criteria.

## 1. Introduction

In modern medical diagnosis or genetic studies, the *receiver operating characteristic* (ROC) curve is a popular tool to evaluate the discrimination performance of a certain biomarker on a disease status or a phenotype. For example, in a continuous-scale test, the diagnosis of a disease is dependent upon whether a test result is above or below a specified cutoff value. Also, genome-wide association studies in human populations aim at creating genomic profiles which combine the effects of many associated genetic variants to predict the disease risk of a new subject with high discriminative accuracy [1]. For a given cutoff value of a biomarker or a combination of biomarkers, the sensitivity and the specificity are employed to quantitatively evaluate the discriminative performance. By varying cutoff values throughout the entire real line, the resulting plot of sensitivity against 1-specificity is a ROC curve. The area under the ROC curve (AUC) is an important one-number summary index of the overall discriminative accuracy of a ROC curve, by taking the influence of all cutoff values into account. Let *Y*_*D*_ be the response of a diseased subject, and let $Y_{\overset{-}{D}}$ be the response of a nondiseased subject; then, the AUC can be expressed as $P(Y_{D}>Y_{\overset{-}{D}})$ [2]. Pepe [3] and Zhou et al. [4] provided broad reviews on many statistical methods for the evaluation of diagnostic tests.

Traditional ROC analyses do not consider the effect of characteristics of study subjects or operating conditions of the test, so test results may be affected in the way of influencing distributions of test measurements for diseased and/or nondiseased subjects. Additionally, although the number of genes is large, there may be only a small number of them associated with the disease risk or phenotype. Therefore, regression models are introduced into the ROC analysis. Chapter Six in Pepe [3] offered a wonderful introduction to the adjustment for covariates in ROC curves. As reviewed in Rodríguez-Álvarez et al. [5], there are two main methodologies of regression analyses in ROC: (1) “induced” methodology, which firstly models outcomes of diseased and nondiseased subjects separately and then uses these outcomes to induce ROC and AUC and (2) “direct” methodology, which directly models the AUC on all covariates. In this paper, we focus on the induced methodology, to which current model selection techniques may be extended.

If there are many covariates, the variable selection issue arises in terms of the consideration of model interpretation and estimability. There are two main groups of variable selection procedures. One is the best-subset selection associated with criteria such as cross-validation (CV, [6]), generalized cross-validation (GCV, [7]), AIC [8], and BIC [9]. The other is based on regularization methods such as LASSO [10], SCAD [11], and adaptive LASSO [12], with tuning parameters selected by the same criteria such as CV and BIC. Procedures in the second group have recently become popular because they are stable [13] and applicable for high-dimensional data [14].

So far, not much attention has been drawn on the topic of variable selection in the ROC regression. Two possible reasons may account for this situation. Firstly, if we model outcomes of diseased and nondiseased subjects separately, selected submodels may be different. The difference will result in difficulties in interpretation, because it is natural to expect that the same set of variables contributes to discriminating diseased and nondiseased subjects. Secondly, most current criteria for variable selection procedures focus on the prediction performance or variable selection consistency. However, in the ROC regression, instead of prediction or model selection, our focus is the precision of an estimated AUC, which means that most popular criteria may not be appropriate. Claeskens and Hjort [15] argued that these “one-fit-all” model selection criteria aim at selecting a single model with good overall properties. Alternatively, they developed the focused information criterion (FIC), which focuses on a parameter singled out for interests. The insight behind this criterion is that a model that gives good precision for one estimand may be worse when used in inference for another estimand. Wang and Fang [16] successfully applied the FIC to variable selection in linear models and demonstrated that the FIC exactly improved the estimation performance of singled-out parameters. This “individualized” criterion exactly fits the ROC regression.

The remaining parts of this paper are organized as follows. In Section 2, we rewrite the ROC regression into a grouped variable selection form so that current criteria can be applied. Then, a general two-stage framework with a BIC selector for the group SCAD under the local model assumption is proposed in Section 3. Simulation studies and a real data analysis are given in Sections 4 and 5. A brief discussion is provided in Section 6. All proofs are presented in the Supplement; see Supplementary Materials available online at http://dx.doi.org/10.1155/2013/436493.

## 2. ROC Regression

In this section, we rewrite the penalized ROC regression with induced methodology into a problem of the grouped variable selection by SCAD. Initially, we require that all covariates be centered at 0 for the consideration of comparability. Also, for notation simplicity, response variables are centered. If not, we can center responses to finish the model selection and then add centers back to evaluate the AUC. By following notations of the local model, which generalizes the commonly used sparsity assumption, homoscedastic regression models for diseased and nondiseased subjects are assumed as follows:

(1) $$\begin{array}{c} y_{D}=z^{T}\theta_{D}+\sigma_{D}\varepsilon_{D}=x^{T}\beta_{D0}+u^{T}\gamma_{D}+\sigma_{D}\varepsilon_{D},\\ y_{\overset{-}{D}}=z^{T}\theta_{\overset{-}{D}}+\sigma_{\overset{-}{D}}\varepsilon_{\overset{-}{D}}=x^{T}\beta_{\overset{-}{D}0}+u^{T}\gamma_{\overset{-}{D}}+\sigma_{\overset{-}{D}}\varepsilon_{\overset{-}{D}}, \end{array}$$

where *x* includes *p* variables added always, *u* includes *q* variables which may or may not be added, *z* = (*x* ^*T*^, *u* ^*T*^)^*T*^, *β*_*D*0_ and $\beta_{\overset{-}{D}0}$ are *p* dimensional vectors, $\gamma_{D}=\delta_{D}/\sqrt{n_{D}}$ and $\gamma_{\overset{-}{D}}=\delta_{\overset{-}{D}}/\sqrt{n_{\overset{-}{D}}}$ are *q* dimensional vectors with *n*_*D*_ and $n_{\overset{-}{D}}$ as sample sizes for diseased and nondiseased groups, respectively, *θ*_*D*_ = (*β*_*D*0_^*T*^, *γ*_*D*_^*T*^)^*T*^ = (*θ*_*D*1_,…, *θ*_*Dd*_)^*T*^ and $\theta_{\overset{-}{D}}={\left(\theta_{\overset{-}{D}1},\ldots ,\theta_{\overset{-}{D}d}\right)}^{T}$ are *d*≜*p* + *q* dimensional vectors, and *ε*_*D*_ and $\varepsilon_{\overset{-}{D}}$ independently follow *𝒩*(0,1). Especially, if $\delta_{D}=\delta_{\overset{-}{D}}=0_{q}$, a sparse model is given. Then, the AUC given *z* can be written as

(2) $$\begin{array}{c} {\text{AUC}}_{z}=\text{Pr}\left(y_{D}\geq y_{\overset{-}{D}}\;|\;z\right)=\Phi \left(\frac{z^{T}\left(\theta_{D}-\theta_{\overset{-}{D}}\right)}{\sqrt{\sigma_{\overset{-}{D}}^{2}+\sigma_{D}^{2}}}\right), \end{array}$$

where Φ(·) is the cumulative distribution function of a standard normal distribution. Clearly, the narrow model is *𝒮*_0_ = {1,…, *p*}, including all constant effects *β*_*D*0_ and $\beta_{\overset{-}{D}0}$. More details of the local model assumption are provided in the following section.

Assume that observed i.i.d that the samples are {(*y*_*Di*_, *z*_*Di*_)}, *i* = 1,…, *n*_*D*_, and $\left\{(y_{\overset{-}{D}j},z_{\overset{-}{D}j})\right\}$, $j=1,\ldots ,n_{\overset{-}{D}}$. Instead of selecting separate models, we consider the following single objective function with a group penalty, given a tuning parameter *λ*:

(3) Qλ(θD,θD−)=12nσD2 ∑i=1nD(yDi−zDiTθD)2+12nσD−2 ∑j=1nD−(yD−j−zD−jTθD−)2+λ∑s=1dpλ(||θs||),

where $\theta_{s}={\left(\theta_{\overset{-}{D}s},\theta_{Ds}\right)}^{T}$, a 2-dimensional vector, with the *s*th component *θ*_*Ds*_ of *θ*_*D*_ and the *s*th component $\theta_{\overset{-}{D}s}$ of $\theta_{\overset{-}{D}}$, and ∂*p*_*λ*_(*w*)/∂*w* = *λI*(*w* ≤ *λ*) + max⁡⁡(0, *aλ* − *w*)*I*(*w* > *λ*)/(*a* − 1) with *a* = 3.7. More generally, instead of the *L*_2_ norm for ***θ***_*s*_, we can define ${||\theta_{s}||}_{K_{s}}=\sqrt{\theta_{s}^{T}K_{s}\theta_{s}}$ with a positive definite 2 × 2 matrix *W*_*s*_. Then, given *λ*, the minimizer of (3) can be obtained as an estimate of ${\left(\theta_{D}^{T},\theta_{\overset{-}{D}}^{T}\right)}^{T}$. The motivation of considering such a penalty on ***θ***_*s*_ jointly rather than separately is that the inclusion or exclusion of the effect of a certain variable should be simultaneous for both diseased and nondiseased groups. It may not be appropriate to include either *θ*_*Dk*_ or $\theta_{\overset{-}{D}k}$ in the model only, which will bring troubles in interpretation of the resulting model. This is exactly the motivation of the group LASSO method by Yuan and Lin [17] to handle categorical variables, and the group SCAD by Wang et al. [18] to address spline bases.

Note that there are two separate summations of residual squares in (3). In order to comply with the framework of selecting grouped variables, a modified version of the objective function (3) is required. Let ⊗ be the Kronecker product operator. Define $\theta =\theta_{\overset{-}{D}}⊗{\left(1,0\right)}^{T}+\theta_{D}⊗{\left(0,1\right)}^{T}$, $\;z_{\overset{-}{D}j}=z_{\overset{-}{D}j}⊗{\left(1,0\right)}^{T}$, $j=1,\ldots ,n_{\overset{-}{D}}$, and **z**_*Di*_ = *z*_*Di*_ ⊗ (0,1)^*T*^, *i* = 1,…, *n*_*D*_. In matrix form, we have

(4) $$\begin{array}{c} Y={\left(y_{\overset{-}{D}1},\ldots ,y_{\overset{-}{D}n_{\overset{-}{D}}},y_{D1},\ldots ,y_{Dn_{D}}\right)}^{T},\\ Z={\left(z_{\overset{-}{D}1},\ldots ,z_{\overset{-}{D}n_{\overset{-}{D}}},z_{D1},\ldots ,z_{Dn_{D}}\right)}^{T}, \end{array}$$

where *Y* is an $n≜n_{\overset{-}{D}}+n_{D}$ dimensional vector with components *y*_*i*_, *i* = 1,…, *n*, and *Z* is an *n* × 2*d* dimensional matrix. Clearly, there are *d* grouped variables, and *Z* can be split into *d* submatrices *Z* = (*Z*_*l*_,…, *Z*_*d*_), each of which includes two consecutive columns of *Z* in turn. Similarly, ***θ*** = (***θ***_1_^*T*^,…, ***θ***_*d*_^*T*^)^*T*^ with $\theta_{m}={\left(\theta_{\overset{-}{D}m},\theta_{Dm}\right)}^{T}$, *m* = 1,…, *d*. Additionally, due to different variances of healthy and diseased subjects, weighted least squares should be applied. Let *W* be a diagonal matrix, with each diagonal entry

(5) $$\begin{array}{c} W_{ii}=\{\begin{array}{cc} \sigma_{\overset{-}{D}}^{-2} & \text{if}\;i=1,\ldots ,n_{\overset{-}{D}},\\ \sigma_{D}^{-2} & \text{if}\;i=n_{\overset{-}{D}}+1,\ldots ,n. \end{array} \end{array}$$

Then, the objective function (3) is written as

(6) $$\begin{array}{c} Q_{\lambda }\left(\theta \right)=\frac{1}{2n}{||Y-\overset{d}{\underset{m=1}{\sum }}Z_{m}\theta_{m}||}_{W}^{2}+\lambda \overset{d}{\underset{m=1}{\sum }}p_{\lambda }\left(||\theta_{m}||\right). \end{array}$$

Furthermore, in order to facilitate computation with current R packages, we would define transformed observations by weighting. Simply, put $\tilde{Y}=W^{1/2}Y$ and ${\tilde{Z}}_{m}=W^{1/2}Z_{m}$. Therefore,

(7) $$\begin{array}{c} Q_{\lambda }\left(\theta \right)=\frac{1}{2n}{||\tilde{Y}-\overset{d}{\underset{m=1}{\sum }}{\tilde{Z}}_{m}\theta_{m}||}^{2}+\lambda \overset{d}{\underset{m=1}{\sum }}p_{\lambda }\left(||\theta_{m}||\right). \end{array}$$

Finally, the penalized ROC regression (3) has been written into a group SCAD-type problem (7). Then, current model selection criteria, like CV, GCV, AIC, and BIC, can be applied to select a final model. For this specific ROC regression problem, where AUC is the focus, these criteria may not be appropriate. Therefore, as argued by Claeskens and Hjort [15], the FIC can play a role here.

Under the local model assumption, a novel procedure of applying the FIC to the grouped variable selection is developed, which is motivated by Wang and Fang [16]. Briefly speaking, the procedure consists of two steps. Firstly, a narrow model, containing variables added always, is identified through the objective function (7). Secondly, the FIC is applied to select a subgroup of remaining variables. As a consequence, the final model is the combination of variables selected in both two steps. Details are provided in the following section. In terms of FIC, naturally, the focus parameter is the AUC at a given *z*_0_; that is, $\mu (\theta )=\Phi \left((z_{0}^{T}⊗(-1,1))\theta /\sqrt{\sigma_{\overset{-}{D}}^{2}+\sigma_{D}^{2}}\right)$ with $\partial \mu /\partial \theta =\phi \left((z_{0}^{T}⊗(-1,1))\theta /\sqrt{\sigma_{\overset{-}{D}}^{2}+\sigma_{D}^{2}}\right)\left(z_{0}⊗{\left(-1,1\right)}^{T}/\sqrt{\sigma_{\overset{-}{D}}^{2}+\sigma_{D}^{2}}\right)$.

Later, in simulation studies, the separate variable selection for diseased and nondiseased models will also be utilized to make a comparison. We expect, the group selection is superior to the separate selection.

## 3. A BIC Selector for Group SCAD under the Local Model Assumption

This section follows notations used in the two fundamental papers of the FIC: Hjort and Claeskens [19] and Claeskens and Hjort [15]. Furthermore, we allow grouped variables, each of which stands for a factor, such as a series of dummy variables coded from a multilevel categorical variable. The starting assumption of the FIC is that some variables are added to the regression model always and the others may or may not be added; that is,

(8) $$\begin{array}{c} y_{i}=x_{i}^{T}\beta_{0}+u_{i}^{T}\gamma +\varepsilon_{i},\;i=1,\ldots ,n, \end{array}$$

where *x*_*i*_ includes *p* variables which are added always, *u*_*i*_ includes *q* variables which may or may not be added, and *ε*_*i*_ ~ *𝒩*(0, *σ*_*ε*_^2^). Without loss of generality, both *x*_*i*_ and *y*_*i*_ are standardized to remove the intercept term. Furthermore, we assume that *x*_*i*_ actually consists of *K* factors, that is, *x*_*i*_ = (*x*_*i*1_^*T*^,…, *x*_*iK*_^*T*^)^*T*^, and the corresponding *β*_0_ = (*β*_01_^*T*^,…, *β*_0*K*_^*T*^)^*T*^, with dimensions *p*_*k*_ for each *x*_*ik*_ and *β*_0*k*_, *k* = 1,…, *K*, such that ∑_*k*=1_^*K*^ *p*_*k*_ = *p*. Similarly, *u*_*i*_ consists of *L* factors, that is, *u*_*i*_ = (*u*_*i*1_^*T*^,…, *u*_*iL*_^*T*^)^*T*^, and the corresponding *γ* = (*γ*_1_^*T*^,…, *γ*_*L*_^*T*^)^*T*^, with dimensions *q*_*l*_ for each *u*_*il*_ and *γ*_*l*_, *l* = 1,…, *L*, such that ∑_*l*=1_^*L*^ *q*_*l*_ = *q*. Let *z*_*i*_^*T*^ = (*x*_*i*_^*T*^, *u*_*i*_^*T*^) = (*z*_*i*1_^*T*^,…, *z*_*iM*_^*T*^), with *d*≜*p* + *q* dimensions, and each *z*_*im*_ has *d*_*m*_ dimensions, *m* = 1,…, *M*, such that *M*≜*K* + *L* and ∑_*m*=1_^*M*^ *d*_*m*_ = *d*. Let *Y* = (*y*_1_,…, *y*_*n*_)^*T*^, *X* = (*x*_1_,…, *x*_*n*_)^*T*^, *U* = (*u*_1_,…, *u*_*n*_)^*T*^, and *Z* = (*z*_1_,…, *z*_*n*_)^*T*^. For simplicity, assume that the residual variance *σ*_*ε*_^2^ is estimated based on the full model and is not considered as a parameter.

In the literature of the variable selection, in order to show the selection consistency of a variable selection procedure, usually, the true model is assumed to be sparse. Thus, the sparsity assumption plays a critical role in the current model selection literature. Many procedures have been shown to be selection consistent under this sparsity assumption [20]. For example, the SCAD with tuning parameter selected via BIC has been shown to be selection consistent by Wang et al. [21, 22], and Zhang et al. [23].

However, it is questionable or too strict to assume that the true model is sparse. It is more reasonable and flexible to consider the local model (8) with *θ*_true_^*T*^ = (*β*_0_^*T*^, *γ* ^*T*^) and $\gamma =\gamma_{0}+\delta /\sqrt{n}$ as a true model, where *γ*_0_ = 0_*q*_ for the purpose of variable selection, under which the FIC is developed. This model is close to the sparse model, but it is different from it by $\gamma -0_{q}=\delta /\sqrt{n}$. The sparsity assumption, with notations in this paper, is equivalent to assume that *δ* = 0_*q*_ and *θ*_true_^*T*^ = (*β*_0_^*T*^, 0_*q*_^*T*^). Therefore, the local model assumption used here is a natural extension of the sparsity assumption. All “consistency” results obtained in this paper still apply to sparse models with grouped variables.

The FIC centers at the inference on a certain estimand or focus, denoted by *μ*_true_ = *μ*(*θ*_true_). It is well known that using a bigger model would typically mean smaller bias but bigger variance. Therefore, the FIC tries to balance the bias and the variance of estimating a certain parameter estimand. To be specific, like what any existing criterion does, among a possible model range, the FIC starts with a narrow model that includes only variables in *x*_*i*_ and searches over submodels including some factors in *u*_*i*_. The whole process leads to totally 2^*L*^ submodels, one for each subset of {1,…, *L*}.

In this framework, various estimators of the focus parameter range from ${\hat{\mu }}_{\text{full}}=\mu ({\hat{\beta }}_{\text{full}},{\hat{\gamma }}_{\text{full}})$ to ${\hat{\mu }}_{\text{narr}}=\mu ({\hat{\beta }}_{\text{narr}},0_{q})$. In general, the FIC attempts to select a subset $\hat{𝒮}$ associated with the smallest mean squared error (MSE) of ${\hat{\mu }}_{𝒮}=\mu ({\hat{\beta }}_{𝒮},{\hat{\gamma }}_{𝒮},\gamma_{0,{𝒮}^{c}})$, where *𝒮* ^*c*^ is the complement of *𝒮* and the subscript *𝒮* means a subset of corresponding vectors indexed by *𝒮*.

### 3.1. Stage 1: Consistent Selection of the Narrow Model

Once assuming the true model (8) with *θ*_true_^*T*^ = (*β*_0_^*T*^, *γ* ^*T*^) and $\gamma =\delta /\sqrt{n}$ as well as grouped variables, here arises the first important question regarding whether we can select the narrow model *𝒮*_0_ = {1,…, *K*} consistently. A similar question has been addressed by Wang and Fang [16], where they considered nongrouped variables. In the following, we show that the group SCAD with a tuning parameter selected via BIC can consistently select the narrow model.

Wang et al. [18] extended the SCAD, proposed by Fan and Li [11], to grouped variables and established its oracle property, following an elegant idea of the group LASSO [17]. The group SCAD generates an estimate via following penalized least squares:

(9) $$\begin{array}{c} {\hat{\theta }}_{\lambda }=\underset{\theta }{\text{argmin}}\left\{\frac{1}{2n}{||Y-Z\theta ||}^{2}+\overset{M}{\underset{m=1}{\sum }}p_{\lambda }\left(||\theta_{m}||\right)\right\}, \end{array}$$

where *θ* = (*θ*_1_^*T*^,…, *θ*_*M*_^*T*^)^*T*^ with *d*_*m*_-dimensional *θ*_*m*_, and *p*_*λ*_(·) is defined in the previous section. Let ${\hat{𝒮}}_{\lambda }=\{m:{\hat{\theta }}_{\lambda m}\neq 0_{d_{m}}\}$ be the selected narrow model for a given *λ*. With similar arguments in the previous section, the *L*_2_ norm used in the penalty can be replaced by any metric with the form ||*θ*_*m*_||_*K*_*m*__≜(*θ*_*m*_^*T*^ *K*_*m*_ *θ*_*m*_)^1/2^ such that *K*_*m*_ is a symmetric *d*_*m*_ × *d*_*m*_ positive definite matrix.

Under the local model assumption with no grouped variables, Wang and Fang [16] showed that, with a tuning parameter *λ* selected via BIC, the SCAD is selection consistent; that is, with probability tending to one, the narrow model can be identified. Similarly, a BIC selector can be defined based on the group SCAD as follows:

(10) $$\begin{array}{c} {\hat{\lambda }}_{\text{B}}=\underset{\lambda }{\text{argmin}}\left\{\log\left({\hat{\sigma }}_{\lambda }^{2}\right)+\frac{{\text{df}}_{\lambda }\log\left(n\right)}{n}\right\}, \end{array}$$

where ${\hat{\sigma }}_{\lambda }^{2}={||Y-Z{\hat{\theta }}_{\lambda }||}^{2}/n$ and ${\text{df}}_{\lambda }=\sum_{m\in {\hat{𝒮}}_{\lambda }}d_{m}$. We expect that the group SCAD is still selection consistent in the sense that $\text{Pr}({\hat{𝒮}}_{{\hat{\lambda }}_{\text{B}}}={𝒮}_{0})\to 1$ as *n* → *∞*, provided that *𝒮*_0_ is the narrow model.

Formally, within the framework of FIC, assuming that the local model (8) is the true model and that *𝒮*_0_ is the narrow model, we show the following theorem. Proofs can be found in the Supplement.

**Theorem 1**

Under some mild conditions (see the Supplement for details), one has that

(11) $$\begin{array}{c} \text{ Pr }\left({\hat{𝒮}}_{{\hat{\lambda }}_{B}}={𝒮}_{0}\right)\to 1,\;as\;\;n\to \infty , \end{array}$$

provided that model (8) with *θ*_true_ = (*β*_0_^*T*^, *γ* ^*T*^)^*T*^ and $\gamma =\delta /\sqrt{n}$ is the true model.

**Remark**

If we assume that *δ* = 0_*q*_, that is, the model is sparse, then Theorem 1 provides a BIC selector for the tuning parameter in the group SCAD, which can consistently identify nonzero effects. In other words, we extend the BIC selector for the SCAD proposed by Wang et al. [21] to the situation with the group SCAD.

Theorem 1 also implies both advantages and disadvantages of the BIC, which have been discussed by Wang and Fang [16]. Briefly speaking, the BIC sacrifices prediction consistency [24] in the sense of filtering all of the variables whose effect sizes are of order $O(1/\sqrt{n})$ to achieve the model selection consistency. The previous theorem provides a data-driven method to consistently specify a narrow model, which is critical before applying FIC. In the following subsection, we suggest a two-stage framework to apply the FIC based upon a narrow model selected via the BIC, in order to recover part of the variables filtered by the BIC.

### 3.2. Stage 2: FIC

In Stage 1, a narrow model, ${\hat{𝒮}}_{0}=\{1,\ldots ,\hat{K}\}$, has been identified via the group SCAD with a tuning parameter selected via BIC. In Stage 2, any subset of ${\hat{𝒮}}_{0}^{c}=\{\hat{K}+1,\ldots ,M=\hat{K}+\hat{L}\}$ can be added to ${\hat{𝒮}}_{0}$. A direct application of the FIC proposed by Claeskens and Hjort [15] is not plausible even for moderate size of $\hat{L}$, because there are $2^{\hat{L}}$ subsets of ${\hat{𝒮}}_{0}^{c}$. Furthermore, the best-subset selection is unstable [13]. Therefore, similar to Wang and Fang [16], without double minimizations through both subsets and tuning parameters proposed by Claeskens [25], we suggest limiting the search domain to those subsets on the solution path from any group regularization procedure such as group LASSO or group SCAD.

With a selected narrow model ${\hat{𝒮}}_{0}=\{1,\ldots ,\hat{K}\}$, let ${\tilde{x}}_{i}={\left(z_{i1}^{T},\ldots ,z_{i\hat{K}}^{T}\right)}^{T}$, ${\tilde{u}}_{i}={\left(z_{i,\hat{K}+1}^{T},\ldots ,z_{iM}^{T}\right)}^{T}$, $\tilde{\beta }={\left(\theta_{1}^{T},\ldots ,\theta_{\hat{K}}^{T}\right)}^{T}$, and $\tilde{\gamma }={\left(\theta_{\hat{K}+1}^{T},\ldots ,\theta_{M}^{T}\right)}^{T}$. Then, a solution path is generated from the following group LASSO procedure (or group SCAD):

(12) $$\begin{array}{c} \left({\hat{\beta }}_{\tau },{\hat{\gamma }}_{\tau }\right)=\underset{\tilde{\beta },\tilde{\gamma }}{\text{argmin}}\left\{\overset{n}{\underset{i=1}{\sum }}\frac{{\left(y_{i}-{\tilde{x}}_{i}^{T}\tilde{\beta }-{\tilde{u}}_{i}^{T}\tilde{\gamma }\right)}^{2}}{2n}+\tau \overset{\hat{L}}{\underset{l=1}{\sum }}||{\tilde{\gamma }}_{l}||\right\}, \end{array}$$

where the tuning parameter *τ* controls the grouped variables included in the subset ${\hat{𝒜}}_{\tau }=\{l:{\hat{\gamma }}_{\tau l}\neq 0_{q_{l}}\}$. As the tuning parameter *τ* varies from some large value to 0, ${\hat{𝒜}}_{\tau }$ increases from an empty set to a “full” set $\left\{1,\ldots ,\hat{L}\right\}$. Then, we utilize the FIC to guide the selection of *τ* in (12) over the resulting ${\hat{𝒜}}_{\tau }$'s, which consist of a search domain.

Now, Stage 2 of the FIC for a certain focus *μ*_true_ = *μ*(*θ*_true_) is summarized as follows. For a given *τ*, a subset ${\hat{𝒜}}_{\tau }$ is provided by indices of nonzero factors from (12). Then, based on the submodel $𝒮={\hat{𝒮}}_{0}\;\cup \;{\hat{𝒜}}_{\tau }$, the FIC_*τ*_ is evaluated according to a formula developed in Claeskens and Hjort [15, formula (3.3)], which is essentially a parametric estimate of the MSE of *μ*_true_ on a model *𝒮*. Consequently, *τ* is selected as

(13) $$\begin{array}{c} {\hat{\tau }}_{\text{F}}=\underset{\tau }{\text{argmin}}\;{\text{FIC}}_{\tau }, \end{array}$$

and the final submodel is selected as ${\hat{𝒮}}_{\text{F}}={\hat{𝒮}}_{0}\cup {\hat{𝒜}}_{{\hat{\tau }}_{\text{F}}}$.

## 4. Simulation

 Simulated data are generated under models (1) with 0 as intercepts. Moderate sample sizes are set to $n_{\overset{-}{D}}=50$ and *n*_*D*_ = 50, compared with 8 and 20 as numbers of covariates. Three scenarios of parameters are considered in the following:

1. $\sigma_{\overset{-}{D}}=\sigma_{D}=2$, *β*_*D*_ = (1.5,2, 3), $\gamma_{Dj}=(3-0.5(j-1))/\sqrt{n_{D}}$, *j* = 1,…, 5, *θ*_*D*_ = (*β*_*D*_^*T*^, *γ*_*D*_^*T*^)^*T*^, $\beta_{\overset{-}{D}}=(0.5,1,2)$, $\gamma_{\overset{-}{D}j}=(1-0.2(j-1))/\sqrt{n_{\overset{-}{D}}}$, *j* = 1,…, 5, $\theta_{\overset{-}{D}}={\left(\beta_{\overset{-}{D}}^{T},\gamma_{\overset{-}{D}}^{T}\right)}^{T}$, *p* = 3, *q* = 5, *d* = 8;
2. $\sigma_{\overset{-}{D}}=\sigma_{D}=2$, *β*_*D*_ = (1.5,2, 3), $\gamma_{Dj}=(2-0.05(j-1))/\sqrt{n_{D}}$, *j* = 1,…, 17, *θ*_*D*_ = (*β*_*D*_^*T*^, *γ*_*D*_^*T*^)^*T*^, $\beta_{\overset{-}{D}}=(0.5,1,2)$, $\gamma_{\overset{-}{D}j}=(1-0.05(j-1))/\sqrt{n_{\overset{-}{D}}}$, *j* = 1,…, 17, $\theta_{\overset{-}{D}}={\left(\beta_{\overset{-}{D}}^{T},\gamma_{\overset{-}{D}}^{T}\right)}^{T}$, *p* = 3, *q* = 17, *d* = 20;
3. $\sigma_{\overset{-}{D}}=\sigma_{D}=1$, *θ*_*Dj*_ = 3/2*j*, $\theta_{\overset{-}{D}j}=2/2j$, *j* = 1,…, 8, *d* = 8.

Clearly, the narrow model of the first two settings is {1,2, 3}, whereas, for the third one, no clear boundary is specified between big effects and small effects.

Corresponding to each setting, test datasets *z*_01_, *z*_02_, and *z*_03_ are selected to generate AUC around 0.6, 0.8, and 0.95 to accommodate low-, moderate-, and high-accuracy cases, respectively. Consider the following:

1. *z*_01_ = (0.2,…, 0.2)^*T*^, AUC = 0.611; *z*_02_ = (0.7,…, 0.7)^*T*^, AUC = 0.838; *z*_03_ = (1.2,…, 1.2)^*T*^, AUC = 0.955;
2. *z*_01_ = (0.15,…, 0.15)^*T*^, AUC = 0.613; *z*_02_ = (0.45,…, 0.45)^*T*^, AUC = 0.805; *z*_03_ = (0.9,…, 0.9)^*T*^, AUC = 0.957;
3. *z*_01_ = (0.3,…, 0.3)^*T*^, AUC = 0.613; *z*_02_ = (0.9,…, 0.9)^*T*^, AUC = 0.806; *z*_03_ = (1.8,…, 1.8)^*T*^, AUC = 0.958.

Besides the proposed two-stage framework (FIC) with group SCAD, for comparison purpose, four popular variable selection criteria, including 5-fold CV, GCV, AIC, and BIC, are also employed. Additionally, the SCAD penalty is applied to diseased and healthy groups separately to show the gain of applying the group SCAD.

Two popular measurements, $\text{MSE}=E{\left(\mu ({\hat{\theta }}_{{\hat{𝒮}}_{\text{F}}})-\mu (\theta_{\text{true}})\right)}^{2}$ and the mean absolute error (MAE), defined by $E|\mu ({\hat{\theta }}_{{\hat{𝒮}}_{\text{F}}})-\mu (\theta_{\text{true}})|$, are utilized to evaluate the prediction performance of selected models based on different criteria, where ${\hat{\theta }}_{{\hat{𝒮}}_{\text{F}}}$ is an estimate of *θ* based on the final model ${\hat{𝒮}}_{\text{F}}$ selected by a certain selection criterion. Due to the limited range of AUC and skewed distributions of estimates of AUC especially at boundaries, the MAE is supposed to be more appropriate.

In this paper, a composite measurement, the F-measure, is employed to evaluate the performance of selecting the narrow model among various methods, including commonly used proportions of selecting underfitting, correct, and overfitting models separately. As noted by Lim and Yu [26], a high F-measure means that both false-positive and false-negative rates are low. Define Precision = true positivity, Recall = true discovery and then, F-measure≜(2 · Precision · Recall)/(Precision + Recall). All results are summarized based on 500 repetitions according to simulation settings in Tables 1, 2, and 3.

Table 1 indicates that the BIC has the best performance to identify the narrow model, compared with others. Also, if there are more weak signals, like Setting 2, the performance is not as good as that of Setting 1. This is reasonable, because, with increasing number of variables given the sample size, it is more challenging to filter weak signals, even under the sparsity assumption. From Table 2, we can see that, in all three settings, these five methods perform well. Specifically, for moderate and large AUC cases, the FIC performs slightly better, providing smaller MAE. Additionally, in these cases, the FIC improves the BIC substantially, which once again indicates that the BIC would filter weak signals.

In order to show how we can benefit from applying the grouped variable selection, separate model selections for diseased and healthy subjects are also considered, and results are summarized in Table 3. By comparing Tables 2 and 3, in most cases, the group penalty provides smaller MSE and MAE for every criterion. Due to limited range of the AUC, all MSE and MAE values in Tables 2 and 3 are small, but the group selection can improve separate selections by as high as 25%. It is not surprising to see that, in high AUC situations, differences are small, and separate selections with BIC are better. Possible reasons are the following: (1) there is no much room for an estimated AUC to vary when it is close to 1; (2) separate selections with BIC offer a larger flexibility to obtain a sparse model.

## 5. Real Data Analysis

In this section, we demonstrate the proposed procedure by the audiology data reported by Stover et al. [27], which has been analyzed by Pepe [3, 28]. The dataset contains results of distortion product otoacoustic emissions (DPOAE) test used to diagnose the hearing impairment. There are 208 subjects who were examined at different combinations of three frequencies (*f*) and three intensities (*L*) of the DPOAE device. An audiometric threshold can be obtained for each combination. At a particular frequency, if the audiometric threshold is greater than 20 dB HL, an ear was classified as hearing impaired. In the original dataset, there are multiple records for each subject. In this study, we randomly select one record for each subject, and among 208 subjects there are 55 subjects with hearing impairment. The test result is the negative signal-to-noise ratio, −SNR. The covariates used in Dodd and Pepe [29] are *z*_*f*_ = frequency Hz/100, *z*_*L*_ = intensity dB/10, and *z*_*D*_ = (hearing threshold − 20) dB/10. In order to encourage the model selection, we incorporate two-way interaction terms. Quadratic terms are not included due to the high correlation between each variable and its quadratic term. Therefore, *z* is the centered (*z*_*f*_, *z*_*L*_, *z*_*D*_, *z*_*f*_ *z*_*L*_, *z*_*f*_ *z*_*D*_, *z*_*L*_ *z*_*D*_)^*T*^ for each element.

Former studies on this dataset showed that −SNR provided quite high discriminative performance and that *z*_*f*_ had a small effect. In order to avoid specifying inappropriate covariates, we randomly select three centered observations from the whole dataset as focused subjects.

Table 4 shows AUC values of models selected by each method as well as corresponding model sizes. CV, AIC, and GCV tend to select a full model. On the contrary, BIC tends to select a sparse model, only containing *z*_*D*_. The full model may not provide the largest AUC, because a large model will bring instability and ruin the AUC. As indicated in the table, for the second test point, both BIC and FIC provide a higher AUC than the full model. But a single variable selected by the BIC seems to be too strict. By focusing on the precision of estimated focus parameter, the FIC provides a customized way to fill the gap: for the first test point, three main effects are selected; for the second one, *z*_*L*_ and *z*_*D*_ are selected; for the third one, only *z*_*D*_ is selected. Based on the precision of estimated AUC, the FIC performs as a compromise, selecting models to generate AUC values in the middle.

## 6. Discussion

In this paper, we rewrite the model selection problem of the ROC regression into a grouped factor selection form with induced methodology. Also, we develop a two-stage framework to apply the FIC to select a final model with group SCAD under the local model assumption. Specifically, if the true model is sparse, our framework naturally accommodates current model selection criteria. Furthermore, the BIC selector is proved to be model selection consistent if either a sparse or a local model is assumed, in the sense of selecting a sparse model or a narrow model.

Most current model selection criteria aim at the prediction performance or model selection consistency; thus, in the ROC regression where the AUC is a focus parameter, they may not be appropriate. This observation motivates an application of FIC, which is shown to perform well through simulation studies. Therefore, our method has a potential application in genetic studies, where the number of gene arrays is always large, compared with the sample size.

For the direct methodology, the literature based on generalized estimating equations is prosperous, which is motivated by the range [0,1] of the AUC, similar to the probability of a binary random variable. Our future work will extend the framework developed here to generalized estimating equations and apply it to the ROC regression with the direct methodology.

As discussed by one referee, it is possible that some coefficients are the same for both *Y*_*D*_ and $Y_{\overset{-}{D}}$. As in (1), modeling them separately will increase the degree of freedom in (3), especially when a large number of genes are covariates. If the shrinkage of a coefficient, which is known a priori to be the same in both diseased and healthy groups, is not necessary, then it is natural for the FIC to include it in the narrow model with a single coefficient. By using the proposed objective function, a fused LASSO type of penalty may be applied to obtain such kind of structure, in addition to the group LASSO/SCAD. Friedman et al. [30] provided a note on the group LASSO and the sparse group LASSO, which could shed light on the question here. It will be also an interesting topic in the future.

## Supplementary Material

In the supplement, we prove the Theorem 1 by establishing 4 key lemmas. This theorem shows the developed BIC selector can consistently identify the narrow model with grouped variables.

## Figures and Tables

**Table 1:** Model selection performance for group SCAD.

Setting	Method	F-measure (%)
1	CV	71.3
	GCV	72.8
	AIC	70.9
	BIC	77.4

2	CV	66.0
	GCV	66.0
	AIC	66.2
	BIC	67.8

**Table 2:** Prediction of AUC at *z*_0*k*_ with group SCAD. Size means the number of selected factors, where each factor contains two variables.

Setting	Methods	*z*_01_			*z*_02_			*z*_03_		
		MSE	MAE	Size	MSE	MAE	Size	MSE	MAE	Size
1	CV	0.00345	0.0467	4.72	0.00295	0.0437	4.72	0.00114	0.0255	4.72
	GCV	0.00345	0.0467	4.49	0.00294	0.0432	4.49	0.00114	0.0251	4.49
	AIC	0.00349	0.0468	4.90	0.00291	0.0428	4.90	0.00110	0.0246	4.90
	BIC	0.00335	0.0461	3.62	0.00317	0.0450	3.62	0.00147	0.0278	3.62
	FIC	0.00339	0.0464	4.23	0.00283	0.0426	4.23	0.00108	0.0247	4.23

2	CV	0.00328	0.0461	8.31	0.00279	0.0428	8.31	0.00073	0.0209	8.31
	GCV	0.00339	0.0470	9.43	0.00281	0.0433	9.43	0.00066	0.0206	9.43
	AIC	0.00344	0.0472	12.05	0.00285	0.0434	12.05	0.00064	0.0204	12.05
	BIC	0.00324	0.0458	6.14	0.00328	0.0462	6.14	0.00129	0.0259	6.14
	FIC	0.00327	0.0459	7.97	0.00290	0.0440	7.97	0.00081	0.0224	7.97

3	CV	0.00369	0.0483	6.67	0.00439	0.0535	6.67	0.00199	0.0317	6.67
	GCV	0.00367	0.0483	6.14	0.00436	0.0533	6.14	0.00197	0.0316	6.14
	AIC	0.00369	0.0484	6.36	0.00441	0.0534	6.36	0.00201	0.0318	6.36
	BIC	0.00367	0.0482	5.14	0.00473	0.0549	5.14	0.00247	0.0345	5.14
	FIC	0.00368	0.0483	5.46	0.00451	0.0532	5.49	0.00219	0.0324	5.49

**Table 3:** Prediction of AUC at *z*_0*k*_ with models on diseased and healthy groups separately. Size means the sum of numbers of selected variables in diseased and non-diseased groups.

Setting	Methods	Size	*z*_01_		*z*_02_		*z*_03_	
			MSE	MAE	MSE	MAE	MSE	MAE
1	CV	8.78	0.00384	0.0490	0.00303	0.0439	0.00107	0.0247
	GCV	8.23	0.00383	0.0488	0.00298	0.0432	0.00103	0.0239
	AIC	8.18	0.00383	0.0488	0.00397	0.0432	0.00102	0.0239
	BIC	6.96	0.00383	0.0490	0.00313	0.0447	0.00114	0.0254

2	CV	14.47	0.00483	0.0566	0.00351	0.0481	0.00079	0.0218
	GCV	16.31	0.00553	0.0609	0.00390	0.0515	0.00069	0.0218
	AIC	15.46	0.00545	0.0606	0.00388	0.0516	0.00069	0.0218
	BIC	12.67	0.00514	0.0590	0.00384	0.0502	0.00092	0.0225

3	CV	12.29	0.00405	0.0494	0.00481	0.0558	0.00225	0.0332
	GCV	10.55	0.00403	0.0497	0.00461	0.0541	0.00208	0.0320
	AIC	10.55	0.00402	0.0497	0.00461	0.0541	0.00209	0.0320
	BIC	9.53	0.00403	0.0499	0.00468	0.0550	0.00206	0.0325

**Table 4:** Estimated AUC at three test points. Size means the number of selected factors.

Methods	Test point 1		Test point 2		Test point 3	
	AUC	Size	AUC	Size	AUC	Size
CV	0.971	6	0.916	6	0.982	6
AIC	0.971	6	0.916	6	0.982	6
GCV	0.971	6	0.916	6	0.982	6
BIC	0.949	1	0.957	1	0.944	1
FIC	0.963	3	0.957	2	0.944	1
