# Supplementary material for: Variable Selection in ROC Regression
Source: Comput Math Methods Med. 2013 Nov 7;2013:436493. doi: 10.1155/2013/436493 (PMC3838845; doi:10.1155/2013/436493)
Supplement: Supplementary file 1 — In the supplement, we prove the Theorem 1 by establishing 4 key lemmas. This theorem shows the developed BIC selector can consistently identify the narrow model with grouped variables. [file 436493.f1.pdf]

Variable Selection in ROC Regression Supplementary Material

Binhuan Wang

New York University School of Medicine, New York NY 10016

September 13, 2013

In the supplement, we will provide the proof of Theorem 1, which directly follows the counterpart in Wang and Fang (2013). In the following, model (3.1) is the true model, with $\theta_{\text{true}} = (\beta_0^T, \gamma^T)^T = (\beta_0^T, \delta^T/\sqrt{n})^T$. Let $L(\theta) = \|Y - Z\theta\|^2/2n$, $Q_\lambda(\theta) = L(\theta) + \sum_{m=1}^M p_\lambda(\|\theta_m\|)$, and $\theta_0 = (\beta_0^T, \mathbf{0}_q^T)^T$. Additionally, assume that $Z^T Z/n \to Q$ as $n \to \infty$. Let $\mathcal{S}_{\text{full}} = \{1, \ldots, M\}$ denote the full model, $\mathcal{S}_0 = \{1, \ldots, K\}$ denote the narrow model, and $\mathscr{A} = \{\mathcal{S} : \mathcal{S} \subset \mathcal{S}_{\text{full}}\}$ be the collection of all submodels of $\mathcal{S}_{\text{full}}$. For any given $\mathcal{S} \in \mathscr{A}$, let $Z_{\mathcal{S}}$ be the $n \times \sum_{m \in \mathcal{S}} d_m$ submatrix of Z consisting of those columns indexed by S, and similarly, we can define $\theta_{\mathcal{S}}$ and $Q_{\mathcal{S}}$. Firstly, based on the work by Wang and Fang (2013), we have following two lemmas.

Lemma 1 *(Wang and Fang, 2013) As $n \to \infty$, $\sqrt{n}\partial L(\theta_0)/\partial\theta \xrightarrow{d} \mathcal{N}(Q_{\mathcal{S}_0}\delta, Q/\sigma_\varepsilon^2)$.*

Lemma 2 *If $\mathcal{S} \supseteq \mathcal{S}_0$, then $\widehat{\sigma}_{\mathcal{S}}^2 \xrightarrow{P} \sigma_{\mathcal{S}}^2 = \sigma_\varepsilon^2$. If $\mathcal{S} \not\supseteq \mathcal{S}_0$, then $\widehat{\sigma}_{\mathcal{S}}^2 \xrightarrow{P} \sigma_{\mathcal{S}}^2 > \sigma_\varepsilon^2$.*

Wang, Chen and Li (2007) showed the oracle property of group SCAD, i.e., $\widehat{\theta}_\lambda = \text{argmin}_\theta\, Q_\lambda(\theta)$ with $\lambda = \lambda_n$ where $\lambda_n \to 0$ and $\sqrt{n}\lambda_n \to \infty$, providing the sparse model $\mathcal{S}_0$ is the true model. We extend it to the local model.

Lemma 3 *Under the same conditions of Theorem 1 in Wang, Chen and Li (2007), except assuming that model (3.1) is the true model with $\gamma = \delta/\sqrt{n}$, if $\lambda_n \to 0$ and $\sqrt{n}\lambda_n \to \infty$, then with probability tending to 1, $\widehat{\mathcal{S}}_{\lambda_n} = \{m : \widehat{\theta}_{\lambda_n m} \neq \mathbf{0}_{d_m}\} = \mathcal{S}_0$.*

Proof of Lemma 3: By Lemma 1, $\sqrt{n}\partial L(\theta_0)/\partial\theta = O_p(1)$. Following proofs of Theorem 1 in Fan and Li (2001) and Theorem 1 in Wang, Chen and Li (2007), we can show that there exists a local minimizer of $\widehat{\theta}$ of $Q_{\lambda_n}(\theta)$ such that $\|\widehat{\theta} - \theta_0\| = O_p(n^{-1/2})$. Note that, for $m = K+1, \ldots, M$,

$$\frac{\partial Q_\lambda}{\partial \theta_m} = \frac{\partial L}{\partial \theta_m} + p'_\lambda(\|\theta_m\|)\frac{2}{\|\theta_m\|}(|\theta_m|\text{sgn}(\theta_m)),$$

where $|\theta_m|\text{sgn}(\theta_m)$ is in componentwise meaning. Furthermore, following the proof of Lemma 1 of Fan and Li (2001), we can show that with probability tending to 1, for any given β^* satisfying $\|\beta^* - \beta_0\| = O_p(n^{-1/2})$ and some constant C, $Q((\beta^{*T}, \mathbf{0}_q^T)^T) = \min_{\|\gamma^*\| \leq Cn^{-1/2}} Q((\beta^{*T}, \gamma^{*T})^T)$. Then Lemma 3 follows. □

Lemma 4 $\Pr(\text{BIC}_{\lambda_n} = \text{BIC}_{\mathcal{S}_0}) \to 1$*, as* $n \to \infty$.

Proof of Lemma 4: Let $\text{BIC}_\lambda = \log(\widehat{\sigma}^2_\lambda) + \text{df}_\lambda \log(n)/n$ (the objective function in (**??**)) and $\text{BIC}_\mathcal{S} = \log(\widehat{\sigma}^2_\mathcal{S}) + \sum_{m\in\mathcal{S}} d_m \log(n)/n$. If λ_n satisfies $\lambda_n \to 0$ and $\sqrt{n}\lambda_n \to \infty$, we have $\|\widehat{\theta}_{\lambda_n} - \theta_0\| = O_p(n^{-1/2})$ (obtained from Lemma 3) and $\Pr(\widehat{\mathcal{S}}_{\lambda_n} = \mathcal{S}_0) \to 1$. Let $\widehat{\theta}^T_{\lambda_n} = (\widehat{\theta}^T_{\lambda_n,\mathcal{S}_0}, \widehat{\theta}^T_{\lambda_n,\mathcal{S}_0^c})$, clearly $\widehat{\theta}_{\lambda_n,\mathcal{S}_0} \to \beta_0 \neq \mathbf{0}_p$ follows. Thus $\Pr(\|\widehat{\theta}_{\lambda_n m}\| > a\lambda_n, m \in \mathcal{S}_0) \to 1$, and a is the constant in the group SCAD penalty. Based on similar techniques in the proof of Lemma 3 of Wang *et al.* (2007), Lemma 4 follows. □

Based on all previous lemmas, it suffices to prove Theorem 1. Let $\Omega_- = \{\lambda \in \Omega : \mathcal{S}_\lambda \nsupseteq \mathcal{S}_0\}$ and $\Omega_+ = \{\lambda \in \Omega : \mathcal{S}_\lambda \supsetneqq \mathcal{S}_0\}$, where Ω is a possible bounded positive range of λ.

Proof of Theorem 1: The proof, similar with Theorem 1 in Wang and Fang (2013), addresses two cases, i.e., lack-of-fit and over-fit.

Case 1: if $\lambda \in \Omega_-$. Lemma 4 shows that, $\text{BIC}_{\lambda_n} = \log\widehat{\sigma}^2_{\mathcal{S}_0} + p\log(n)/n$ with probability 1, and Lemma 2 shows, $\text{BIC}_{\lambda_n} \to \log(\sigma^2_\varepsilon)$ in probability. Since $\widehat{\sigma}^2_\lambda \geq \widehat{\sigma}^2_{\widehat{\mathcal{S}}_\lambda}$ by the meaning of OLS estimates, $\text{BIC}_\lambda \geq \log(\widehat{\sigma}^2_{\widehat{\mathcal{S}}_\lambda}) \geq \min_{\{\mathcal{S}:\mathcal{S}\nsupseteq\mathcal{S}_0\}} \log(\widehat{\sigma}^2_\mathcal{S}) \to \min_{\{\mathcal{S}:\mathcal{S}\nsupseteq\mathcal{S}_0\}} \log(\widehat{\sigma}^2_\mathcal{S}) > \log(\sigma^2_\varepsilon)$, where the last inequality follows from Lemma 2. Hence, $\Pr(\inf_{\lambda\in\Omega_-}\text{BIC}_\lambda > \text{BIC}_{\lambda_n}) \to 1$.

Case 2: if $\lambda \in \Omega_+$. Given any $\mathcal{S}^* \supsetneqq \mathcal{S}_0$ with $\text{df}_\lambda = \sum_{m\in\mathcal{S}^*} d_m = d^*$, $\text{SSE}_{\mathcal{S}_0} - \text{SSE}_{\mathcal{S}^*} = Y^T(H_{\mathcal{S}^*} - H_{\mathcal{S}_0})Y$ follows non-central chi-square distribution $\sigma^2_\varepsilon\chi^2_{d^*-p}(\theta^T_{\text{true}}Z'(H_{\mathcal{S}^*} - H_{\mathcal{S}_0})Z\theta_{\text{true}}) = \sigma^2_\varepsilon\chi^2_{d^*-p}(\delta'U'(H_{\mathcal{S}^*} - H_{\mathcal{S}_0})U\delta/n)$, where the last equality follows from projection properties of $H_{\mathcal{S}^*}$ and $H_{\mathcal{S}_0}$. Therefore $\text{SSE}_{\mathcal{S}_0} - \text{SSE}_{\mathcal{S}^*} = O_p(1)$.

Again based on the simple fact $\widehat{\sigma}_\lambda^2 \geq \widehat{\sigma}_{\widehat{\mathcal{S}}_\lambda}^2$ and Lemma 4, $\text{BIC}_\lambda - \text{BIC}_{\lambda_n} \geq \log(\widehat{\sigma}_{\widehat{\mathcal{S}}_\lambda}^2) - \log(\widehat{\sigma}_{\mathcal{S}_0}^2) + (\text{df}_\lambda - p)\log(n)/n$ with probability tending to 1. With standard Taylor expansion technique, $\log(\widehat{\sigma}_{\widehat{\mathcal{S}}_\lambda}^2) - \log(\widehat{\sigma}_{\mathcal{S}_0}^2) = \widehat{\sigma}_{\mathcal{S}_0}^{-2}(\text{SSE}_{\widehat{\mathcal{S}}_\lambda} - \text{SSE}_{\mathcal{S}_0})/n + O_p((\text{SSE}_{\widehat{\mathcal{S}}_\lambda} - \text{SSE}_{\mathcal{S}_0})^2/n^2)$. Hence, with probability tending to 1,

$$n(\text{BIC}_\lambda - \text{BIC}_{\lambda_n}) \geq \widehat{\sigma}_{\mathcal{S}_0}^{-2}(\text{SSE}_{\widehat{\mathcal{S}}_\lambda} - \text{SSE}_{\mathcal{S}_0}) + (\text{df}_\lambda - p)\log(n) + o_p(1).$$

Finally, with probability tending to 1, $\inf_{\lambda\in\Omega_+} n(\text{BIC}_\lambda - \text{BIC}_{\lambda_n}) \geq \widehat{\sigma}_{\mathcal{S}_0}^{-2} \min_{\mathcal{S}\supsetneqq\mathcal{S}_0}(\text{SSE}_{\mathcal{S}} - \text{SSE}_{\mathcal{S}_0}) + \log(n) + o_p(1) = \log(n) + O_p(1)$. Therefore, $\Pr(\inf_{\lambda\in\Omega_+}\text{BIC}_\lambda > \text{BIC}_{\lambda_n}) \to 1$.

By combining results in previous two cases, we prove $\Pr(\inf_{\lambda\in\Omega_-\cup\Omega_+}\text{BIC}_\lambda > \text{BIC}_{\lambda_n}) \to 1$. Consequently, Theorem 1 follows. □
